# A Taguchi-based study on the control factors of reinforced composites with the fiber of coir and pineapple leaves

**DOI:** 10.1016/j.heliyon.2024.e40947

**Published:** 2024-12-06

**Authors:** Md Firoz Kabir, Md Alamgir Hossain, Md Nazmus Sakib, Md Waliullah Shadhin, Md Ariful Alam

**Affiliations:** a Department of Industrial and Production Engineering, Bangladesh Army University of Science and Technology (BAUST), Saidpur, Cantonment, Bangladesh; b Department of Mechanical Engineering, Hajee Mohammad Danesh Science and Technology University (HSTU), Dinajpur, 5200, Bangladesh

**Keywords:** Coir fiber, Pineapple leaf, Mechanical properties, Taguchi orthogonal array analysis, Regression analysis, ANOVA

## Abstract

The use of composite materials, whether metallic or non-metallic, is becoming more popular nowadays because of some of their superior characteristics compared to the use of wood and metallic materials alone. From this perspective, a new natural fiber reinforced composite by varying the fiber orientation was developed in this study using coir and pineapple leaf fiber. This work uses the Taguchi method to investigate the different effects of control factors on mechanical and physical characteristics of the fabricated natural fiber-based composites. Various control factors were used, including fiber ratios, angles of orientation, and mat types. The testing was conducted in accordance with ASTM standards, and the results were validated through various statistical analyses including Taguchi orthogonal array analysis, confirmation tests, regression analysis, and analysis of variance (ANOVA). Based on the analysis and validation, the highest mean impact strength was found 53.93 J/cm^2^, tensile strength 31.94 MPa, flexural strength 46.365 MPa, Rockwell hardness number 77, and lower water absorption rate only 3.62 %. From the confirmation test, margin of errors was found to be 4.84 %, 2.59 %, 2.35 %, 6.62 %, and 2.334 % for impact, tensile, and flexural strength, Rockwell hardness, and water absorption test respectively. The variation of the experimental and predicted results was observed from the regression analysis, and it was 2.93 to 0.4 J/cm^2^, 2.12 to 0.79 MPa, 3.54 to 0.33 MPa, 2.33 to 0.8 RHN, and 0.92 %–0.13 % for impact, tensile, and flexural strength, Rockwell hardness, and water absorption test respectively. Overall, ANOVA analysis was used to examine the effects of different control variables, and it was discovered that the angle of orientation of fibers had a substantial impact on flexural strength and water absorption rate were 72.30 % and 70.89 % respectively. Similarly, mat types on tensile strength and Rockwell hardness with 46.47 % and 50.67 % respectively. In addition, the impact strength was most significantly affected by the wt.% ratio of fibers, which was approximately 50.32 %. From the above characteristics and their environmentally friendly behavior, these composites can be used in the place of synthetic fiber-based products.

## 1 Introduction

Using naturally generated fibers from plants as an efficient reinforcing material for composite structures has proven an effective technique utilized by different peoples throughout history, making it far from a novel concept. In the past, various materials have been recognized for their ability to enhance rigidity, durability, and ability to withstand the force of a particular substance. In recent decades, there has been a growing emphasis on utilizing various types of naturally occurring fiber to enhance the strength of polymer matrices [1]. Extensive experiments are being carried out to investigate the characteristics of composites. There are various methods that can be utilized to convert natural fibers into composite. The goal of these treatments is to optimize the final composites' overall performance, strengthen the fibers' compatibility with the matrix, and improve the fibers' characteristics [2].

The sources of pineapple leaf fiber (PALF), where they appear in plants, and how they are extracted are used to categorize PLF. Compared to other vegetable fibers, PALF is thought to have a better texture. It aids in restoring the climate and by stopping soil erosion, soil quality [3]. The value additions of pineapple leaf (PALF) are often overlooked and underutilized. This waste has the potential to be repurposed into composites that are applicable to a wide range of fields, including biomedical, automotive, furniture, packaging, and infrastructure. These applications can also address environmental concerns and enable farmers to sustain their livelihoods while preserving ecological balance [4]. When it comes to agricultural waste into useable products [5], PALF stands out with its impressive cellulose content of 70–80 %. This makes it an excellent choice for enhancing polymer matrices and achieving a favorable balance between mechanical properties and weight [6]. For engineering and structural applications, the most cost-effective example of lighter weight bio-composite material is pineapple leaf fiber and flour, which are widely available [7]. Coir fiber is an important material because it is versatile, sustainable, eco-friendly, and provides economic benefits to many people around the world [8]. Coir is a highly adaptable substance that is obtained from the outer layer of coconuts. It is known for its strength and has gained significant interest for its ability to enhance polymer strength and increase bulk density, resulting in more affordable composite products [9]. Coir fibers possess a lower cellulose content of 36–43 % and hemicellulose content of 15.17 %, while their lignin content is higher at 32.25 %. Additionally, these fibers have a higher microfibrillar angle ranging from 30 to 45° compared to other fibers. Pineapple leaf fiber possesses remarkable physical and mechanical properties, making it a promising option for use as reinforcing infill bio-composites [10]. Using orthogonal arrays, the Taguchi approach uses fewer tests to analyses a greater number of variables. By utilizing orthogonal arrays, it becomes feasible to greatly decrease the number of experimental parameters. Research and development expenses are also reduced since results drawn from a limited number of trials hold true for the whole experimental area when several experimental variables are simultaneously examined [11].

Mittal et al. [12] carried out an experimental investigation to look at the mechanical characteristics and biological degradation of hybrid epoxy composites that were reinforced with fiber from coir and pineapple leaf. A total of twenty-three composite specimens were analyzed. The specimens were split into two groups: the untreated group and the alkali-treated group. Several tests were conducted to assess tensile, flexural, impact, and weight loss properties. The results showed that, compared to the epoxy composite made entirely of pineapple leaves, the hybrid composites showed a quicker fall in mechanical strength in the natural soil environment. The researchers utilized PALF and coir fibers in an epoxy thermoset to improve its mechanical strength and encourage biodegradability. The incorporation of glass fiber into the Coir-Epoxy composite led to a notable improvement in its mechanical properties. Using SLS/coir fibers to reinforce polyester matrix composites, Arul et al. [13] investigated the materials' mechanical properties. The mechanical characteristics that were examined were tensile, flexural, and impact strengths. In addition, a thorough analysis is conducted on the composites' resistance to breakdown. A simple manual lay-up procedure was used to insert the fibers into the matrix resin at varying weight percentages consisting of 25/75, 50/50, and 75/25, while maintaining a total fiber weight percentage of 30 % throughout the production process. Testing was conducted in accordance with ASTM regulations. After examining the data analysis, it became clear that the hybrid composite of (50/50) stood out compared to other combinations due to its exceptional mechanical properties. Combining both fibers instead of using pure polyester resin shows promising signs of enhancing the mechanical characteristics of resin matrix. Naik et al. [14] examined the duplex stainless steel 2205's tensile strength and welding optimization parameters using tungsten inert gas welding. The analysis of variance and Taguchi method serve as the foundation for the debate. The Taguchi approach of orthogonal L9 design experiment is used to minimize the error that happens in the neural network during output prediction and to detect the problem that arises during the welding process using an orthogonal array. Neural networks are mathematical prediction models for optimization, and they are based on the back propagation technique. ANOVA is an approach to statistical analysis that is highly useful for decision-making, enabling us to identify and comprehend variations in process parameters. It also helps determine the ideal number of factors to use in confirmation experiments, which are used to verify the optimal design parameters.

The present study included the fabrication of composites using coir and PALF. In addition, they experienced the inspection in accordance with the ASTM standard to find out the best combination of control factors that would provide outstanding mechanical performance for the composites. Utilizing the confirmation test, the margin of error was measured, and the variation of the experimental and predicted results was then observed using regression analysis. Finally, the ANOVA analysis was performed to investigate the effects of different control variables on the fabricated composites.

## 2 Materials and methodology

### 2.1 Materials

**Fig. 1:**
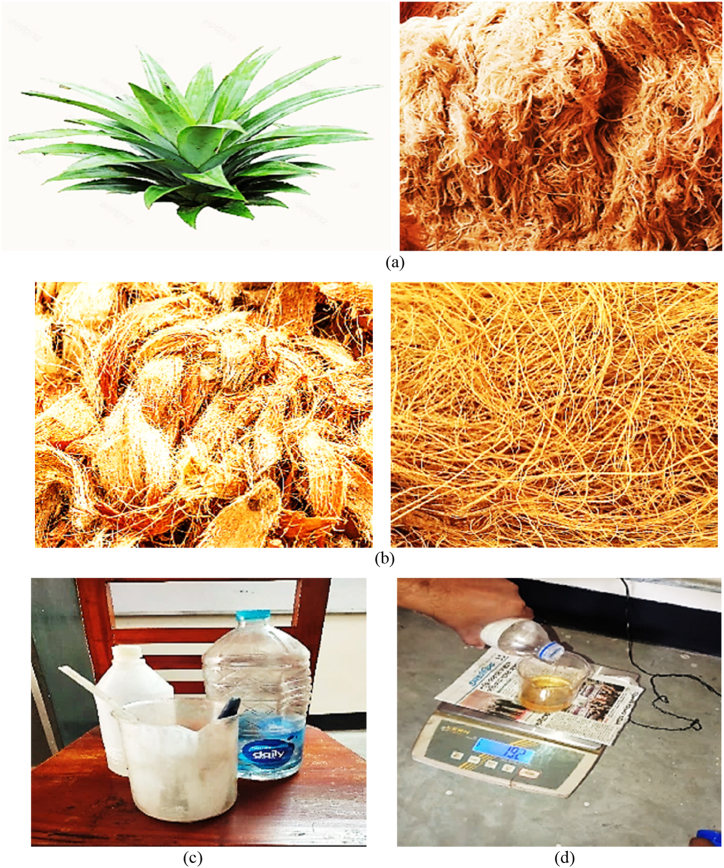
Materials in composite; (a) pineapple leaf and fiber; (b) coir fiber; (c) DGEBA- LY 556 epoxy; (d) TETA- HY 951 hardener.

Natural resources of coir fiber and PALF are shown in Fig. 1(a) and **1(b)**, were selected for the reinforcement in the composite and collected from BSCIC, Tangail, Bangladesh. The epoxy resin with a medium viscosity, Di-glycidyl Bisphenol acid (LY 556), and hardener, Tri-ethylene Tetraamine acid of HY 951, shown in Fig. 1(c) and **(d)**, respectively, were selected as matrix material. To increase strength and improve interfacial adhesion, a hardener was used.

### 2.2 Experimental design

Utilizing the Taguchi technique, optimization was completed for fiber-reinforced composites by identifying the optimal set of variables such as-mechanical characteristics, structural integrity, and performance of the composite material. In this research, three control parameters were implemented for further investigation of the fabricated composites. These are.

i. Fiber ratio.
ii. Angle of orientation of fiber.
iii. Mat types (see Table 1).

**Fig. 2:**
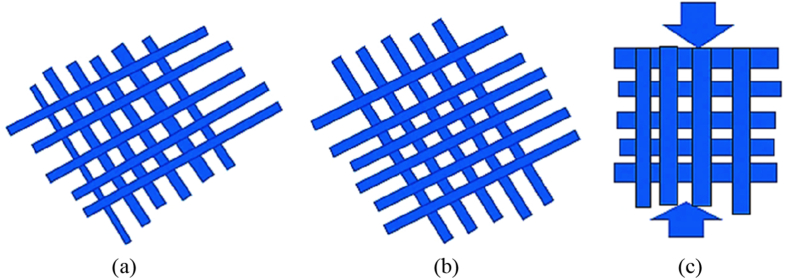
Angle of orientation of fiber; (a) 15°, (b) 30°, and (c) 90°.

The weight percentages of the fibers-matrix and resin-hardener were determined to be 3:7 and 3:1, respectively. The fiber ratio between the pineapple leaf fiber (GSM of 283.68 g/m^2^) and coir fiber (GSM of 507.49 g/m^2^) were 1:1, 1:2 and 2:1 respectively, shown in Table-2. 15°, 30° and 90° were selected as varying angels of orientation of the samples shown in Fig. 2, that were cut using CNC machine (see Table 3).

**Table-1:** Some previous works on fiber consolidated hybrid composites.

Authors	Aim of the work	Findings of the work
Panthapulakkal et al. [15]	Characterization of Mechanical, water-absorbing, and thermal characteristics of composites reinforced with a hybrid combination of hemp and glass fibers.	Adding glass fiber to the hemp polypropylene composite enhanced its mechanical strength and thermal stability.
Haq et al. [16]	Creation of an advanced hybrid composite including soybean oil and UPE.	After incorporating EMS as an alternative (10 %) for UPE and reinforcing it with 1.5 % nano clay, a remarkable beneficial hybrid material was achieved, exhibiting a perfect balance between stiffness and toughness.
Shahzad et al. [17]	Examine the fatigue characteristics and effects of composites reinforced with hemp and glass fibers.	The pure hemp fiber composite showed improved impact and fatigue strength when combined with glass fiber.
Davoodi et al. [18]	Compared the general performance of GMT material for automobile bumper beams with a kenaf/glass epoxy composite.	There is a great deal of promise for using the double hat profile (DHP) hybrid epoxy composite reinforced with glass fiber and kenaf in tiny automotive bumper beams.
Aji et al. [19]	Investigate the Impact of hybridization on the water absorption characteristics and mechanical attributes of HDPE composites reinforced with Kenaf/PALF fibers	Following the hybridization process with PALF, the tensile and flexural characteristics of the Kenaf/HDPE composite were enhanced. The best mechanical properties are found in an HDPE matrix with equal volumes of PALF and Kenaf.
Jawaid et al. [20]	Examine the Jute/EFB-Epoxy hybrid composites' physical and chemical resistance characteristics.	Increasing jute fiber percentage resulted in reduced voids and improved chemical stability in the hybrid composite.
Bhagat et al. [21]	Investigated the mechanical and physical characteristics of epoxy composites using a Coir/Glass hybrid.	The hybrid composite that is reinforced with 20 wt percent glass fiber and 10 wt percent coir fiber (15 mm length) has the highest mechanical strength.
Cheng et al. [22]	Investigating the properties of composites composed of polyester reinforced with Glass/Coir fibers.	After adding glass fabric, COIR-Polyester composite flexural, impact, and modulus of elasticity increased 419 %, 562 %, and 708 %.
Atiqah et al. [23]	Examining the mechanical characteristics of composites composed of polyester-reinforced Kenaf and Glass fibers.	The blended material consists of glass fiber and alkali-treated kenaf fiber in a 15/15 vol ratio is stronger in tension, bending, and impact tests than the other hybrid formulas.
Chevali et al. [24]	Observe the Changes in physical characteristics of ABS polymer with sunflower hull and distiller dry grain fiber loading.	ABS polymer lost some of its tensile, flexural, and impact strength when SFH and DDGS fillers were added. The reason was the inadequate compatibility between the polymer and reinforcing additives.
Yahaya et al. [25]	Impact of the quantities of kenaf fiber present on the physical characteristics of hybridized epoxy-based materials made of kenaf and Kevlar.	When the kenaf fiber concentration increased from 22 % towards 68 %, the void content increased as well, rising from 3.15 % to 25.67 %.
Teja et al. [26]	Impact of SiC filler filling on the mechanical features of a polymer strengthened with sisal fibers	In comparison to the untreated composite material, the sisal-polyester matrix loaded with SiC demonstrates a tensile strength that is 2.53 times larger and an impact strength that is 1.73 times higher.
Arumugaprabhuet al. [27]	Investigating the mechanical properties of composites made from Palmyra and Coir fibers reinforced with polyester.	The addition of Coir fiber to the pure composite resulted in a significant increase in its tensile, flexural, and impact strength. The hybridization ratio of 40:60 (Palmyra/Coir) proved to be effective in enhancing the overall performance of the composite.
Shrivastava et al. [28]	Creating a composite material by combining Coir and Glass fibers with epoxy resin	The addition of glass fiber to a coir-based composite greatly improved its mechanical properties, leading to considerable improvement.
Khalil et al. [29]	Investigating the impact of coconut pulp nano-filled materials on the mechanical characteristics of kenaf/coconut composites containing fibers.	The tensile, flexural, and impact strengths of kenaf/coconut composites made from fibers increase with the addition of coconut pulp nano-filled materials up to 3 wt percent.
Bakri et al. [30]	Investigating the impact of varying amounts of fiber materials in the mechanical characteristics of epoxy-based materials strengthened with Coir/Angustifolia Haw Agave fibers.	Compared to the two mixed compositions (Coir: Agave = 10:20 and 20:10). The incorporation of a 15:15 v/v ratio of Coir to Agave in the composite material leads to enhanced tensile strength, as well as increased tensile and flexural modulus.
Nurazzi et al. [31]	Impact of fibers mixing on the mechanical characteristics of polymer composites enhanced with sugar palm and glass fibers	Greater tensile, flexural, and compressive strengths are shown by the blended composite material, which has a 40-wt percent total fiber content and the same amount of each type of fiber
Rihayat et al. [32]	Explored the mechanical characteristics of hybrid materials strengthened with the fibers of Bamboo, PALF, and coconut.	In comparison with individual materials, hybrid material made with bamboo, PALF, and coconut fiber has a better mechanical property.
Siakeng et al. [33]	Exploring the physical characteristics of composite materials consolidated with PALF/COIR fibers along with PLA.	The incorporation of PALF via COIR/PLA materials resulted in a reduction in their density, ability to absorb water, and height enlargement.
Senthikumar et al. [34]	To find out the optimal machining parameters for Inconel 718 alloy in milling operation.	For maximizing MRR and minimizing tool wear HSN2 is preferable, and for minimizing the rough surface, tinalox coated tool is better option than the other.
Sangilimuthukumar et al. [35]	To investigate the different characteristics of Kevlar-Hemp and Kevlar-Pineapple leaf fiber-based hybrid composites with different weaving architectures.	The twill weave type Kevlar-Pineapple leaf fiber and Kevlar-Hemp fiber-based hybrid composites exhibited better energy absorption and peak load than all types of composites.

**Table-2:** Experimental design of the composites.

Composite specimens	Fiber ratio	Angle of orientation	Mat types
1	1:1	30	Single mat
2	1:1	15	Combine mat (cp-cp)
3	1:1	90	Combine mat (cc-pp)
4	1:2	30	Combine mat (cp-cp)
5	1:2	15	Combine mat (cc-pp)
6	1:2	90	Single mat
7	2:1	30	Combine mat (cc-pp)
8	2:1	15	Single mat
9	2:1	90	Combine mat (cp-cp)

**Table-3:** Matrix material properties [12,36].

Properties	Epoxy of DGEBA- LY 556	Hardener of TETA- HY 951
Appearance	colorless	Brownish yellow
Viscosity @ordinary temp.	9000–12000 mPa's	500–1000 mPa's
Density @ordinary temp.	1130–1160 kg/m^3^	946 kg/m^3^
Molecular weight (*M*_*n*_)	2300 g/mol	146.23 g/mol

**Fig. 3:**
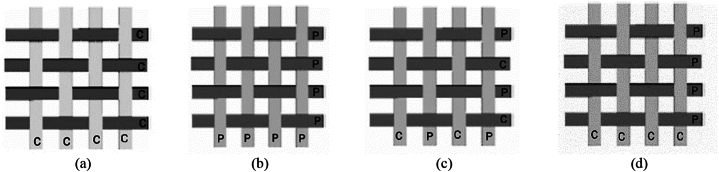
Types of mats; (a) single mat (cc); (b) single mat (pp); (c) combined mat (cp-cp); and (d) combined mat (cc-pp).

Single mat (cc/pp) expressed as S, combined mat (cp-cp) as C2, and (cc-pp) as C1 were taken as another control parameter shown in Fig. 3, during the fabrication of the composites.

Finally, the experiments for two hybrid bio-composites consisting of (3 × 3) L9 orthogonal array with three levels of design are shown in Table-2, including the fiber ratio, angle of orientation of fiber, and mat types.

### 2.3 Fabrication process

**Fig. 4:**
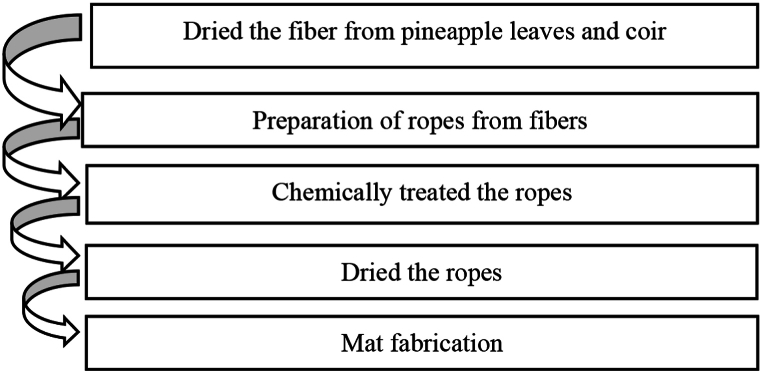
Fabrication of mats.

**Fig. 5:**
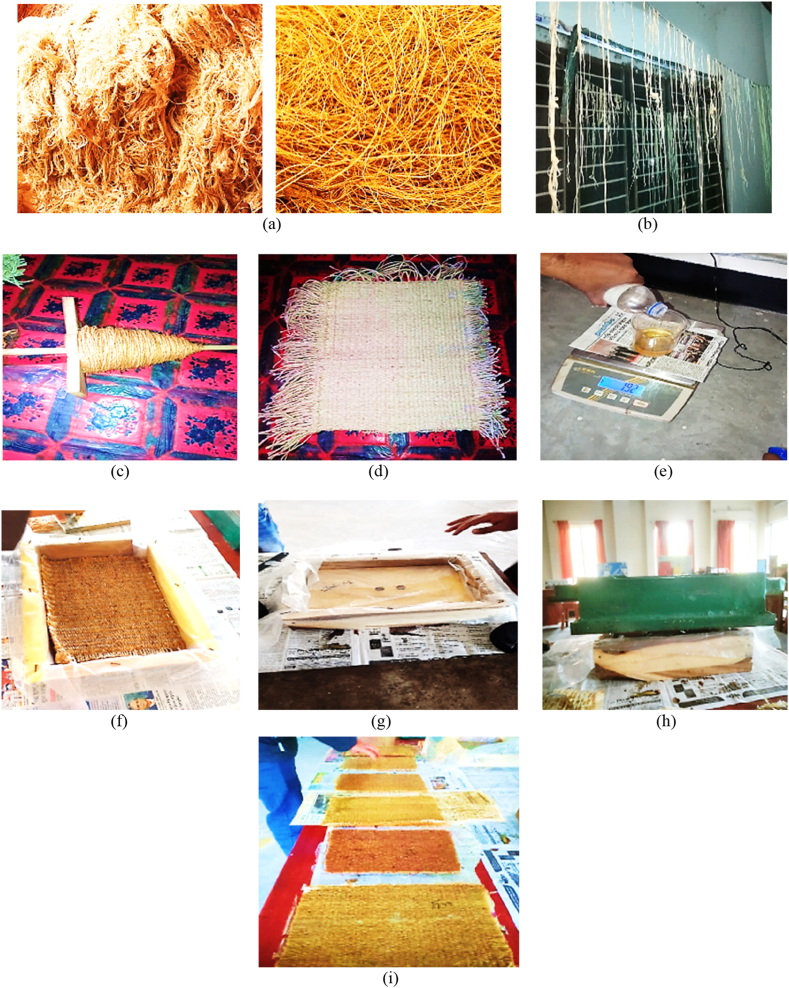
Fabrication process; (a) natural fibers; (b) short yarn from fibers; (c) rope from fibers; (d) fabricated mat; (e) mixing of matrix materials; (f) mats into the mold; (g) covering the mold; (h) load on the mold; and (i) fabricated composites.

The manual lay-up method, which is cheap, requires little work, and doesn't take long time, was used to make composites. First, the fibers shown in Fig. 5(a), were taken from the outside shell of the coir and leaves from pineapple plant. Secondly, they were treated at various steps such as cleaning, washing, and drying to eliminate impurities, moisture, and unwanted substances. Thirdly, the processed short fibers shown in Fig. 5(b), were first twisted to make long fibers like a yarn or rope shown in Fig. 5(c). The fibers were then submerged in a 5 % NaOH solution for a whole day. Next, the fibers were taken out and dried in the sun for a day. Finally, 350 mm × 250 mm x 4 mm dimension's mats shown in Fig. 5(d), were fabricated using a hand loom machine by varying the fiber ratio, angle of orientation, and mat types. The entire sequential process of mats fabrication is depicted in Fig. 4.

For initiating the curing process, a curing agent i.e. hardener of TETA-HY 951 was mixed with the epoxy resin of DGEBA-LY 556 at 1:3 ratio, shown in Fig. 5(e).

The molds were made of wood and to facilitate the composite's removal, a polythene was wrapped to the mold's exterior shown in Fig. 5(f). Then, the matrix with mat was poured at 7:3 ratio into the mold shown in Fig. 5(g). The resin was then evenly applied on the mat using a hand brush and roller. To eliminate any air gaps, a wooden plate was lastly placed over the composite shown in Fig. 5(h). After that, it was allowed to solidify for 48 h. Finally, the composite material was taken out of the mold, its jagged edges were carefully trimmed, shown in Fig. 5(i). The natural fiber composites were then cured by being exposed to ordinary air.

### 2.4 Mechanical characterization

#### 2.4.1 Impact strength

**Fig. 6:**
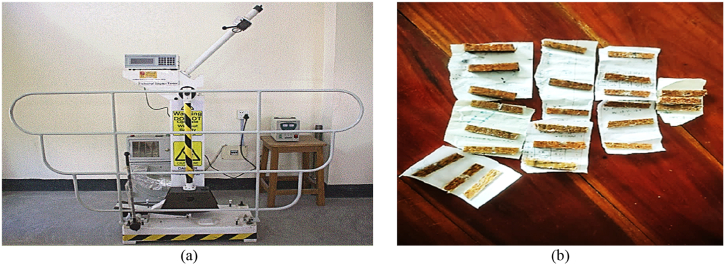
Impact strength testing apparatus; (a) HSM55 pendulum impact tester; (b) specimens for impact test.

A material's ability to endure energy up to its breaking point is determined by the impact test. The Charpy v-notch test is widely recognized as the go-to method for assessing a material's energy absorption during fracture. By the standards set of the ASTM D256, the specimens endured impact strength testing using the HSM55 pendulum impact tester depicted in Fig. 6(a). A set of three test specimens, each of 130 mm × 27 mm with three different thicknesses, were created, as depicted in Fig. 6(b).

#### 2.4.2 Tensile strength

**Fig. 7:**
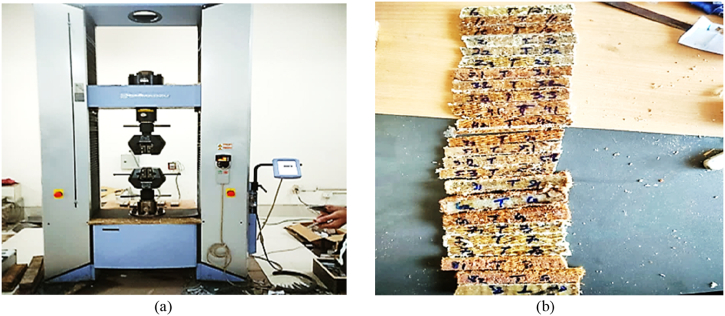
Tensile strength testing apparatus; (a) WANCE HUT A106 UTM; (b) specimens for tensile strength.

It refers to the force that pulls a material like wires, ropes, or beams, until it breaks. The specimens in this study were fabricated with dimensions of 280 mm × 20 mm with three different thickness. These specimens were then subjected to testing following the ASTM D3039/3039M criteria. Fig. 7(b) illustrates the specimens for tensile testing and Fig. 7(a) depicted the WANCE HUT A106 universal testing machine for conducting the tensile tests.

#### 2.4.3 Flexural strength

**Fig. 8:**
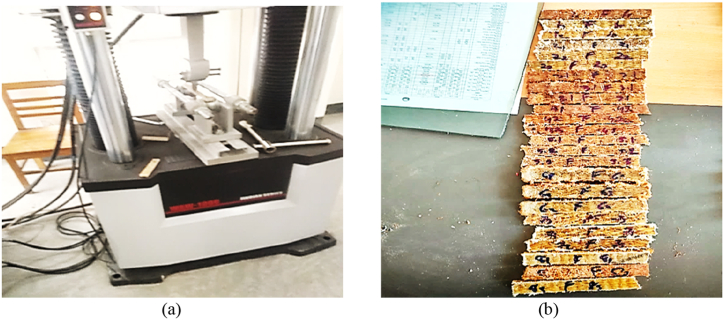
Flexural strength testing apparatus; (a)WANCE HUT A106 UTM; (b) specimens for flexural strength.

The largest amount of bending force a material can withstand without breaking is known as its flexural strength. A popular method for determining flexural strength is the three-point flexural test, which involves subjecting the material to transverse bending. The WANCE HUT A106 universal testing machine, shown in Fig. 8(a) was utilized to measure the flexural strength of each test specimen following the ASTM D790 criteria. During the fabrication of the composite specimens, three different thicknesses were utilized with 280 mm × 20 mm dimensions, as depicted in Fig. 8(b).

#### 2.4.4 Rockwell hardness

**Fig. 9:**
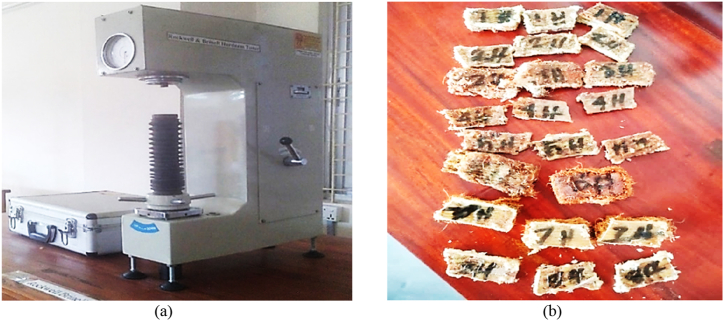
Rockwell hardness testing apparatus; (a) HSM51-Rockwell hardness tester; (b) specimens for Rockwell hardness test.

Hardness refers to the ability of a material to withstand indentation or scratching when subjected to a load. The HSM51-Rockwell hardness tester, as depicted in Fig. 9(a), was used to determine the hardness. During the experiment, a diamond indenter was used to apply pressure on the surface of composites. The specimens are displayed in Fig. 9(b) with dimensions of 20 mm × 20 mm for three different thickness.

#### 2.4.5 Water absorption test

**Fig. 10:**
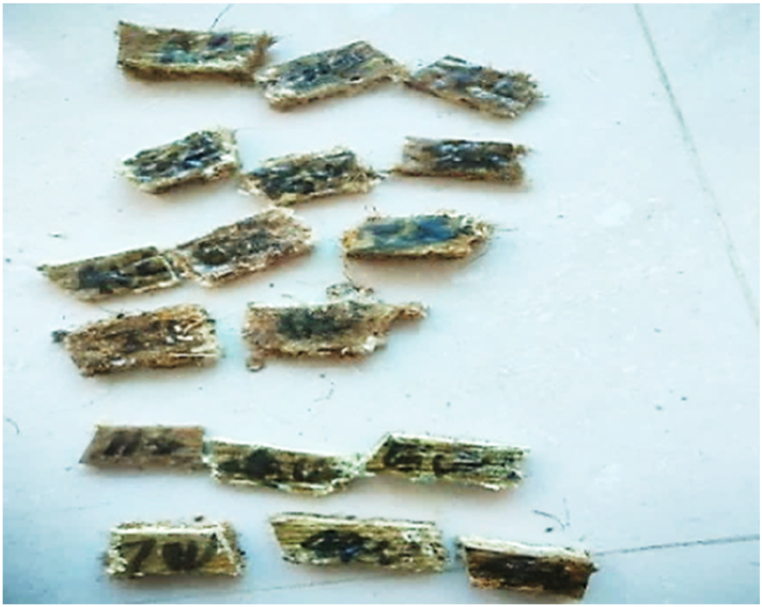
Water-absorption test specimens.

According to ASTM D5229M − 12, water absorption tests were conducted on PALF and coir fiber composites by immersing them in distilled water at room temperature, as shown in Fig. 10. Using an accurate balancing machine, the samples were regularly pulled out and their surface water content immediately measured to determine the amount of water absorbed. Conversely, after 24, 48, and 72 h, the samples were regularly weighed. The following formula was then employed to determine the water absorption based on the weight difference:

$$\text{Water}\;\text{absorption}\;\text{rate}\;\left(\%\right)=\frac{\text{weight}\;\text{of}\;\text{wet}\;\text{sample}-\text{weight}\;\text{of}\;\text{dry}\;\text{sample}}{\text{weight}\;\text{of}\;\text{dry}\;\text{sample}}\times 100\%$$

## 3 Result analysis

### 3.1 Impact strength

A material's ability to withstand energy when a sudden force is applied is measured by its impact strength, sometimes referred to as impact resilience. A set of three test specimens of 130 mm × 27 mm, dimensions were created which were tested using the ASTM A370 standard.

**Fig. 11:**
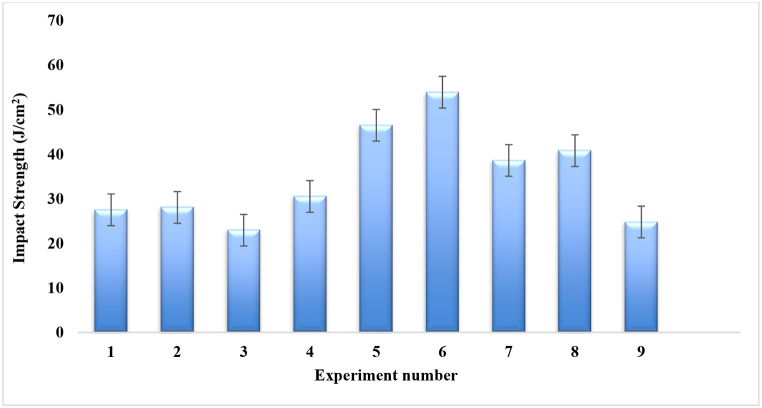
Impact strength comparison for different experiment numbers.

Table-4 shows the nine experimental results of impact strength obtained from 27 samples that were fabricated based on the Taguchi L9 method by varying the compositions of three parameters. The maximum mean impact strength of 53.93 J/cm^2^ was found for experiment number 6 that is very much larger than the recycling of industrial discarded waste [37], in which the fiber ratio was 1:2, angle of orientation was 90° and mat type was single. And minimum impact strength of 22.96 J/cm^2^ was found for experiment number 3, in which the fiber ratio was 1:1, angle of orientation was 90° and mat type was combined (cc-pp). From Table-4 and Fig. 11, It was also noticed that 1:2 fiber ratio had greater effect on the impact strength rather than 1:1 and 2:1.

**Table-4:** Average experimental impact strength under different control factors.

Expt. No.	Fiber ratio	Angle of orientation	Mat-type	Impact Strength (J/cm^2^)			
				Sample 1	Sample 2	Sample 3	Mean
1	1:1	30	Single mat	35.8	19.2	27.6	**27.53**
2	1:1	15	Combine Mat (cp-cp)	43.4	24.0	16.8	**28.06**
3	1:1	90	Combine Mat (cc-pp)	21.4	25.6	21.9	**22.96**
4	1:2	30	Combine Mat (cp-cp)	34.7	31.3	25.6	**30.53**
5	1:2	15	Combine Mat (cp-cp)	35.0	42.3	62.2	**46.5**
6	1:2	90	Single mat	38.5	41.5	81.8	**53.93**
7	2:1	30	Combine Mat (cc-pp)	28.5	23.3	64.0	**38.6**
8	2:1	15	Single mat	42.4	40.6	39.4	**40.8**
9	2:1	90	Combine Mat (cc-pp)	22.3	26.2	25.9	**24.8**

#### 3.1.1 Taguchi orthogonal array analysis for impact strength

One of the main goals of this investigation is to determine the combination at which the composite will exhibit its highest impact strength. A responsible table of mean impact strength for different control factors is shown below.

**Fig. 12:**
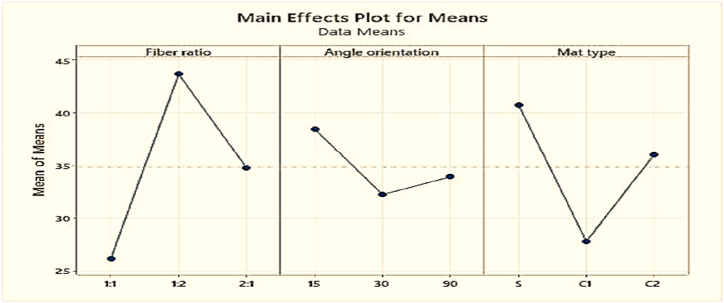
Composite's mean impact strength.

Based on Table-5 and Fig. 12, It was discovered that the fabricated composites were largely influenced by the changes of fiber ratio. Impact strength increases when the coir fiber increases and decreases when the coir fiber decreases. The second crucial component was the mat type, which must be maintained in single mat and the angle of orientation was the third crucial factor which was to be kept at 15° for better strength.

**Table-5:** Response in mean impact strength at various levels of control factors.

Level	Fiber ratio	Angle of orientation	Mat type
1	26.18	**38.45**	**40.75**
2	**43.59**	32.16	27.07
3	34.07	33.23	36.02
Delta	17.41	6.29	13.68
Rank	1	3	2

#### 3.1.2 Confirmation test for impact strength

To validate experimental results, it is essential to conduct a confirmation test, which is strongly advised by Taguchi. The optimal set of variables, according to the Taguchi design of experiments is A (1:2), B (15) and C (single mat). The following equation gives the maximum predicted impact strength [38].

ImpactStrength(Predicted)=A(1:2)+B(15)+C(singlemat)–3Y

=(43.59+38.45+40.75)−(3x34.87)

$$=18.18\;J/{\text{cm}}^{2}$$

At their optimal levels, impact strength averages out to A, B, and C, with Y being the overall mean.

A (1:2). B (15) and C (single mat) are the best combination of control parameters for maximal impact strength. The combination mentioned above led to the creation of a new sample that was used to support the expected impact strength using Taguchi technique. The experimental impact strength was found to be 17.3 J/cm^2^, and the percentage of error was 4.84 %, that is almost half compared to 7.21 % [39].

#### 3.1.3 Regression analysis for impact strength

The impact strength of composite with PALF and coir fiber may be calculated using the following formula:

$$\text{Impact}\;\text{Strength}\;\left(J/{\text{cm}}^{2}\right)=34.86-8.67\left(A\right)+3.60\left(B\right)+1.16\;\left(C\right)$$

**Fig. 13:**
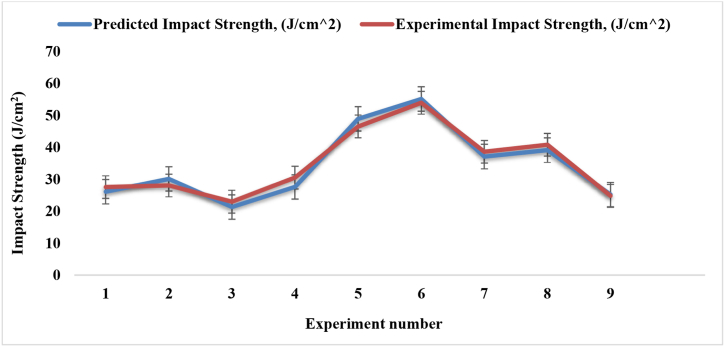
Impact strength comparison between experimental and predicted values.

Fig. 13 indicates the graphical relationship between experimental and predicted impact strength. The two graphs in the figure are almost the same. For experiment numbers 1, 3, 6, 7, 8 and 9, the experimental impaction values are almost assimilated with the predicted impact strength values. Experiment number 2, 4 and 5, the experimental impact strengths are quite near to the predicted impact strength.

#### 3.1.4 ANOVA for impact strength

Table-6 is the general linear model of ANOVA, in which the impact strength of the fabricated composites is primarily determined using the fiber ratio, with a maximum percentage contribution of 50.32 %. The F-distribution table provides the critical value, F (critical) = 7.71 for numerator 1 and denominator 4. The greatest percentage of contribution has a forecasted F-value of 3.48, that is less than 7.71. The P-value, which is higher than 5 % and indicates that the assumption is acceptable, is 0.223, or 22.3 %. Likewise, the angle of orientation of the composite shows the least amount of contribution, at 6.86 %. In this case, the estimated F-value of 0.47 doesn't meet the crucial value of 7.71. Additionally, the P-value, at 0.679, or 67.9 %, is higher compared to 5 %, indicating a statistically substantial chance of adopting the assumptions. In this investigation, the total effect of unaccounted-for control variables yields an error rate of 14.48 %.

**Table-6:** ANOVA for 95 % confidence level of impact strength.

Source	DF	Adj SS	Adj MS	F-Value	P-Value	Contribution
Fiber ratio	2	457.87	228.93	3.48	0.223	**50.32 %**
Angle of orientation	2	62.43	31.21	0.47	0.679	6.86 %
Mat type	2	257.90	128.95	1.96	0.338	28.34 %
Error	2	131.76	65.88			14.48 %
Total	8	909.96				100.00 %

### 3.2 Tensile strength

Tensile strength refers to the highest load that the substance can endure without fracturing when subjected to stretching forces. In this work, a set of three test specimens measuring 280 mm × 20 mm, were fabricated and tested using the ASTM D3039/3039M standards.

**Fig. 14:**
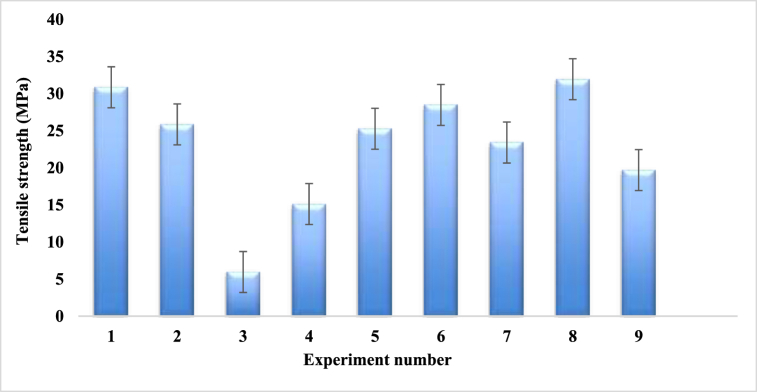
Tensile strength comparison for different experiment numbers.

Table-7 and Fig. 14 reveal the mean tensile strength of the composites which were fabricated by using Taguchi L9 orthogonal array. For experiment number 8, where fiber ratio, angle of orientation and mat type are 2:1, 15 and single mat respectively, shows the maximum tensile strength value of 31.94 MPa, which is very much lower than Kevlar [40,41], palm, kenaf bast, bamboo and banana fiber, individually [42] but higher than the jute fiber woven mat reinforced epoxy composites [43] and kenaf fiber with banana fiber reinforced composite material [44]. On the contrary, experiment number 3 provides the minimum mean tensile strength value of 5.95 MPa, where fiber ratio, angle of orientation and mat type of the composite are 1:1, 90, combine mat (cc-pp) respectively. From Table 7 and Fig. 14, it is noticed that a single mat i.e. experiments number 1, 6 and 8, has the superiority for improving the tensile strength rather than the combined mat. This is the opposite pattern compared to the jute and basalt fiber reinforced composite [45].

**Table-7:** Average experimental tensile strength under different control factors.

Expt. No.	Fiber ratio	Angle of orientation	Mat-type	Tensile Strength (MPa)			
				Sample 1	Sample 2	Sample 3	Mean
1	1:1	30	Single mat	30.8	28.61	32.15	**30.53**
2	1:1	15	Combine Mat (cp-cp)	21.8	32.3	23.46	**25.85**
3	1:1	90	Combine Mat (cc-pp)	4.32	8.09	5.44	**5.95**
4	1:2	30	Combine Mat (cp-cp)	10.09	9.65	25.6	**15.13**
5	1:2	15	Combine Mat (cp-cp)	23.78	19.38	32.62	**25.26**
6	1:2	90	Single mat	25.2	29.67	30.56	**28.47**
7	2:1	30	Combine Mat (cc-pp)	18.93	23.41	27.87	**23.4**
8	2:1	15	Single mat	26.19	30.23	39.4	**31.94**
9	2:1	90	Combine Mat (cc-pp)	17.62	18.55	22.9	**19.69**

A comparison of tensile properties of sisal/hemp green epoxy hybrid bio-composites with this work has been shown below. From the comparison, it is noticeable that tensile strength of these two different works is almost same at higher range.

**Table:** 

Composites pattern	HHHH	HSHS	SHHS	SSSS	HSSH	HHSS	Ref.
Tensile Strength (MPa)	32.0 ± 1.39	31.76 ± 0.88	30.76 ± 1.17	30.70 ± 0.60	30.24 ± 0.73	30.00 ± 1.23	[46]
	31.94	30.13	28.67	25.85	25.26	23.40	This Work
Experiment number	8	1	6	2	5	7	

#### 3.2.1 Taguchi orthogonal array analysis for tensile strength

One of the primaries aims of this work is to determine the optimal combination at which the composite material will demonstrate its maximum tensile strength. Table-8 displays the average tensile strength at various levels of control factors.

**Table-8:** Response in mean tensile strength at various levels of control factors.

Level	Fiber Ratio	Angle Orientation	Mat Type
1	22.95	23.02	**30.31**
2	20.78	**27.68**	20.22
3	**25.01**	18.04	18.20
Delta	4.23	9.65	12.11
Rank	3	2	1

**Fig. 15:**
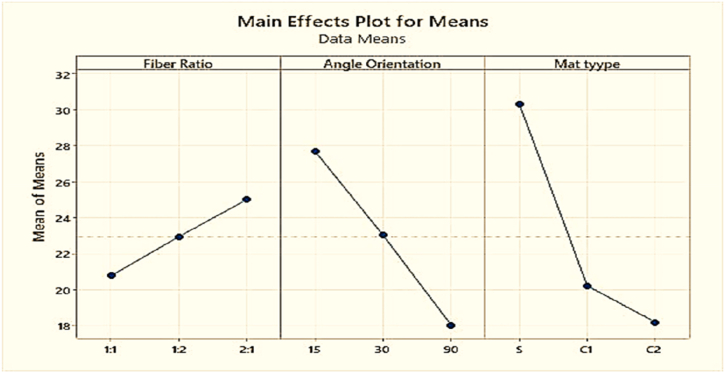
Composite's mean tensile strength.

Based on values of Table-8 and Fig. 15, it is discovered that the tensile strength is highly affected by mat type. A single mat is a layered mat of PALF and coir fiber. The second important factor is the angle of orientation of the composite fibers that should be kept to 15° to 30°. And the least important factor is the fiber ratio that has less effect on the improvement of tensile strength of the composites.

#### 3.2.2 Confirmation test for tensile strength

The optimal set of variables, according to the Taguchi design of experiments, is A (2:1), B (15), C (Single mat). The maximal tensile strength may be calculated using the formula below [38]:

TensileStrength(Predicted)=A(2:1)+B(15)+C(Singlemat)–3Y

=(25.01+27.68+30.31)–(3x22.91)

=14.27MPa.

At their optimal levels, tensile strength averages out to A, B, and C, with Y being the overall mean.

There was no trial in the orthogonal array that corresponded to the ideal combination of control parameters for maximum tensile strength, A (2:1), B (15), C (Single mat). So, a new experiment was done for the verification of the predicted value. The experimental tensile strength was found to be 13.9 MPa, and the percentage of error was 2.59 %, that is little bit higher compared to 1.918 % [39].

#### 3.2.3 Regression analysis for tensile strength

The tensile strength of composite materials with PALF and coir fiber may be calculated using the formula.

TensileStrength(MPa)=22.91−2.10(A)+4.77(B)+7.40(C)

**Fig. 16:**
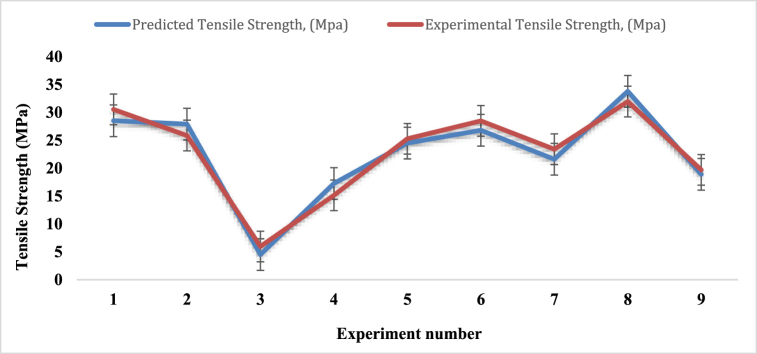
Tensile strength comparison between experimental and predicted values.

The graphical link between the experimental and predicted tensile strengths is displayed on Fig. 16. The two graphs in the figure are almost the same. For experiment numbers 3, 5 and 9, the experimental tensile values are almost assimilated with the predicted tensile strength values. On the other hand, for experiment numbers 1, 2, 4, 6, 7 and 8, the experimental values are quite near to the predicted tensile strength.

#### 3.2.4 ANOVA for tensile strength

Table-9 is the general linear model of ANOVA, in which the tensile strength is primarily determined through the mat type, with a maximum percentage contribution of 46.47 %. The F-distribution table yields the critical value F (critical) = 7.71 for numerator 1 and denominator 4. The greatest percentage of contribution has a forecasted F-value of 2.03, that is less than 7.71.0.330, or 33 % of P value is higher than 5 % and suggests that the prediction is reasonable. Similarly, reinforced composite's fiber ratio displays the lowest level of contribution, with a value of 4.95 %. In this instance, the F value, 7.71 is not met by the predicted F-value of 0.22. Furthermore, the P-value is greater than 5 % at 0.822, or 82.2 %, suggesting a statistically significant for accepting the prediction.

**Table-9:** ANOVA for 95 % confidence level of tensile strength.

Source	DF	Adj SS	Adj MS	F-Value	P-Value	Contribution
Fiber Ratio	2	26.89	13.44	0.22	0.822	4.95 %
Angle of orientation	2	139.64	69.82	1.12	0.471	25.69 %
Mat type	2	252.54	126.27	2.03	0.330	**46.47 %**
Error	2	124.40	62.20			22.89 %
Total	8	543.46				100.00 %

### 3.3 Flexural strength

Flexural strength, which is the stress in a material immediately prior to yielding in a flexure test. In this work, the test specimens with dimensions of 280 mm × 20 mm were tested according to ASTM D790 standards.

**Fig. 17:**
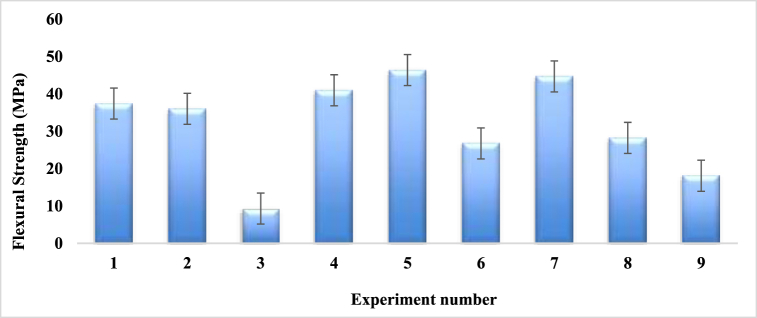
Comparison of flexural strength for different experiment numbers.

The flexural strength of the composites found from the Taguchi L9 orthogonal array are plotted in Table-10**.** Experiment number 5 shows the highest value of 46.365 MPa that is almost half of the flexural strength of jute fiber woven mat reinforced epoxy composites [43], and identical to sugar palm fiber [46], where fiber ratio, angle of orientation and mat type are 1:2, 15 and combine mat (cp-cp) respectively. On the contrary, experiment number 3 provides the minimum flexural strength value of 9.29 MPa, where weight percentage ratio of fiber, angle of orientation and mat type in the composite are 1:1, 90, combine mat (cc-pp) respectively. From Table-10 and Fig. 17, it is noticeable that, for obtaining the maximum strength, the angle of orientation of the fibers should be maintained between 15° to 30°.

**Table-10:** Average experimental flexural strength under different control factors.

Expt. No.	Fiber ratio	Angle of orientation	Mat-type	Flexural Strength (MPa)		
				Sample 1	Sample 2	Mean
1	1:1	30	Single mat	35.35	39.45	**37.4**
2	1:1	15	Combine Mat (cp-cp)	43.34	28.65	**35.95**
3	1:1	90	Combine Mat (cc-pp)	8.81	9.78	**9.29**
4	1:2	30	Combine Mat (cp-cp)	29.10	52.82	**40.96**
5	1:2	15	Combine Mat (cp-cp)	46.29	46.44	**46.365**
6	1:2	90	Single mat	28.64	24.83	**26.735**
7	2:1	30	Combine Mat (cc-pp)	41.68	47.65	**44.66**
8	2:1	15	Single mat	26.19	30.23	**28.21**
9	2:1	90	Combine Mat (cc-pp)	17.62	18.55	**18.08**

#### 3.3.1 Taguchi orthogonal array analysis for flexural strength

Increasing flexural strength is one of the aims of this work. Table-11 shown below is the flexural strength responsible table at various levels of control parameters.

**Table-11:** Response in mean flexural strength at various levels of control factors.

Level	Fiber ratio	Angle of orientation	Mat type
1	27.56	36.86	30.78
2	**38.02**	**41.01**	31.68
3	30.32	18.04	**33.44**
Delta	10.46	22.97	2.66
Rank	2	1	3

**Fig. 18:**
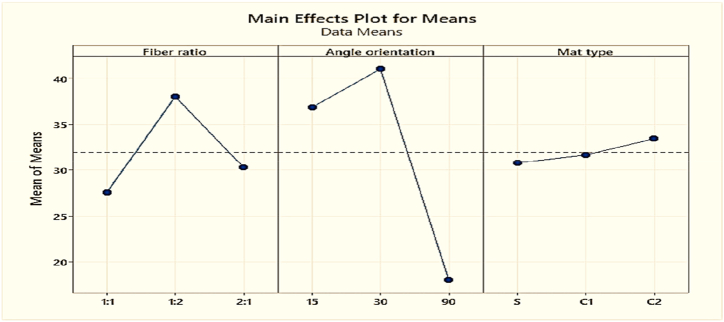
Composite's mean flexural strength.

Based on the numerical values of Table-11 and Fig. 18, the angle of orientation of the fibers in composites is the prime reason for the maximum strength, and 30° angle of orientation has a greater effect on strength. The second important factor is the fiber ratio which should be maintained 1:2 at the weight percentage of PALF and coir fiber.

#### 3.3.2 Confirmation test for flexural strength

The optimal set of variables, according to the Taguchi design of experiments is A (1:2), B (30), C (Combine Mat (cc-pp)). The highest possible flexural strength may be calculated using the following formula [38]:

FlexuralStrength(Predicted)=A(1:2)+B(30)+C(CombineMat(cc−pp))–3Y

=(38.02+41.01+33.44)–(3x31.96)

=16.59MPa.

At their optimal levels, flexural strength averages out to A, B, and C, with Y being the overall mean, for the highest flexural strength.

There was no trial in the orthogonal array that corresponded to the ideal combination of control parameters of A (1:2), B (30), C (Combine Mat (cc-pp)). So, a new experiment was done for the verification of the predicted value incorporating the Taguchi method. The experimental flexural strength was found to be 16.2 MPa, and the percentage of error was 2.35 %, that is very much lower compared to 5.15 % [39].

#### 3.3.3 Regression analysis for flexural strength

The following is the regression formula for calculating the composite's flexural strength with PALF and coir fiber:

FlexuralStrength(MPa)=31.97+6.05(A)+9.04(B)+1.47(C)

**Fig. 19:**
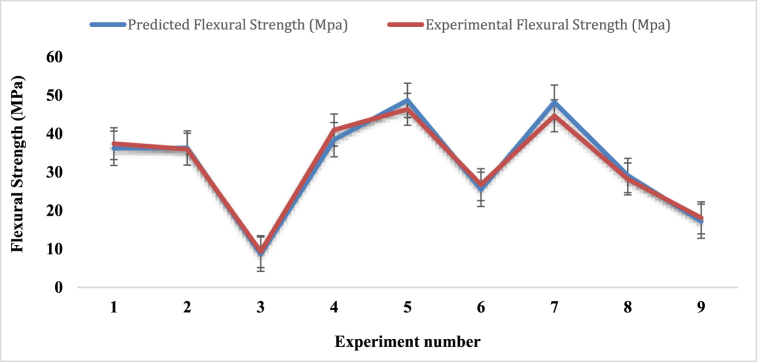
Flexural strength comparison between experimental and predicted values.

Fig. 19 indicates the graphical relationship between experimental and predicted flexural strength. The two graphs in the figure have nearly identical natures. For experiment numbers 2, 3, 6, 8 and 9 the experimental flexural values are almost assimilated with the predicted flexural strength values. For experiment numbers 1, 4, 5 and 7, the values that were seen in the experiment are quite near the results that were expected.

#### 3.3.4 ANOVA for flexural strength

Table-12 is the general linear model of ANOVA, in which the impact strength of the fabricated composites is primarily determined by the angle of orientation, with a maximum percentage contribution of 72.30 %. The F-distribution table yields the critical value F (critical) = 7.71 for numerator 1 and denominator 4. The highest contribution has a predicted F-value of 5.72, that is less than 7.71.0.149, or 14.9 % of P value is higher than 5 % and suggests that the assumption is reasonable. Similarly, the reinforced composite's mat type displays the lowest level of contribution, with a value of 0.88 %. In this instance, the F value, 7.71, is not met by the predicted F-value of 0.07. Furthermore, the P-value is greater than 5 % at 0.935, or 93.5 %, suggesting a statistically significant for accepting the prediction.

**Table-12:** ANOVA for 95 % confidence level of flexural strength.

Source	DF	Adj SS	Adj MS	F-Value	P-Value	Contribution
Fiber ratio	2	176.23	88.116	1.12	0.471	14.17 %
Angle of orientation	2	898.99	449.493	5.72	0.149	**72.30 %**
Mat type	2	10.99	5.493	0.07	0.935	0.88 %
Error	2	157.17	78.586			12.64 %
Total	8	1243.38				100.00 %

### 3.4 Rockwell Hardness

The penetration depth of an indenter under a major load relative to the penetration made by a minor load is measured by the Rockwell test. Specimens with the dimension of 20 mm × 20 mm were tested by incorporating the hardness tester machine of HSM51 (P A. Hilton Ltd., Hampshire, England). The required load was selected by rotating the knob, and the proper diamond indenter (1200) was positioned. The work surface of the machine was set up after the sample had been cleaned. The capstan wheel was turned to bring the indenter tip into contact with the test specimen. The C scale was used for the testing. During the experiment, pressure was applied to the surface of the composites using a diamond-shaped indenter [[47], [48], [49]].

**Fig. 20:**
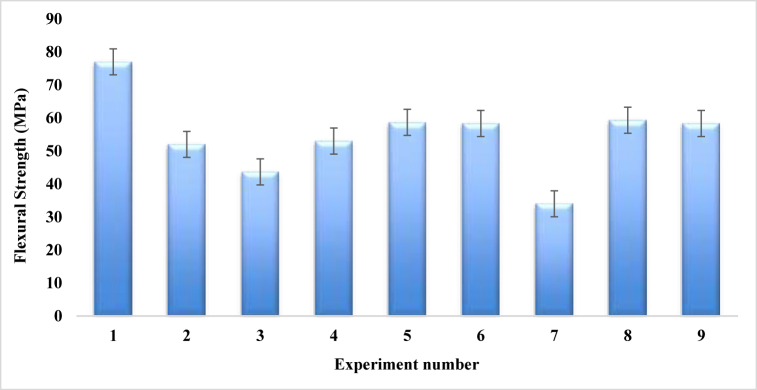
Comparison of Rockwell hardness for different experiment numbers.

Table-13 and the graph in Fig. 20, indicating the Rockwell hardness values corresponding to the experiment number which were fabricated by using Taguchi L9 orthogonal array. Experiment number 1 shows the maximum Rockwell hardness value of 77, where fiber ratio, angle of orientation and mat type are 1:1, 30 and single mat respectively. On the contrary, experiment number 7 provides the minimum Rockwell hardness value of 34, where weight percentage ratio of fiber, angle of orientation and mat type of composites are 2:1, 30, Combine Mat (cc-pp) respectively. It is worth noting that the combined mat typically has an average Rockwell hardness number of around 55, whereas a single mat can reach a maximum Rockwell hardness number of 77. So, it can be said that for the variation of Rockwell hardness, mat types have a great impact on the composites.

**Table-13:** Average experimental Rockwell harness under different control factors.

Expt. No.	Fiber ratio	Angle of orientation	Mat-type	Rockwell Hardness			
				Sample 1	Sample 2	Sample 3	Mean
1	1:1	30	Single mat	72	76	83	**77**
2	1:1	15	Combine Mat (cp-cp)	47	52	57	**52**
3	1:1	90	Combine Mat (cc-pp)	39	42	50	**43.66**
4	1:2	30	Combine Mat (cp-cp)	55	46	58	**53**
5	1:2	15	Combine Mat (cp-cp)	56	62	58	**58.67**
6	1:2	90	Single mat	63	59	53	**58.33**
7	2:1	30	Combine Mat (cc-pp)	29	34	39	**34**
8	2:1	15	Single mat	57	58	63	**59.33**
9	2:1	90	Combine Mat (cc-pp)	58	62	55	**58.33**

#### 3.4.1 Taguchi orthogonal array analysis for rockwell hardness

The larger the number of Rockwell hardness obtained from the experiment is better the approach, because it is one of the main objectives of this work that to maximize the mechanical strength of the composites. Table-14 displays the Rockwell hardness responsible table for different control factors.

**Table-14:** Response in mean Rockwell hardness number at various levels of control factors.

Level	Fiber ratio	Angle of orientation	Mat type
1	**57.56**	**56.67**	**64.89**
2	56.67	54.67	54.44
3	50.55	53.44	45.45
Delta	7	3.22	19.44
Rank	2	3	1

**Fig. 21:**
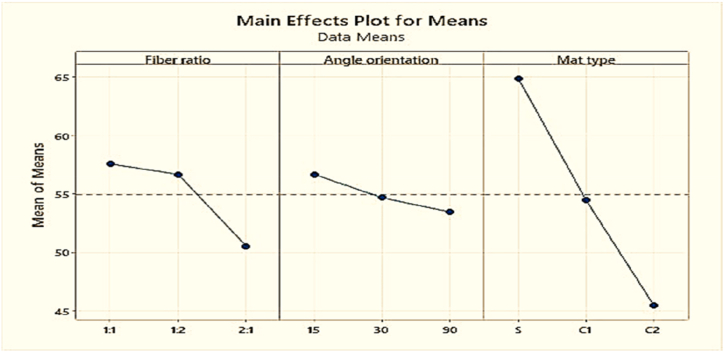
Composite's mean Rockwell hardness number.

Based on Table-14 and Fig. 21, it was discovered that the Rockwell hardness is highly affected by Mat type. Among the variations of mat types, single mat that is layered of coir and PALF showed the best Rockwell hardness number. The other two factors, i.e. fiber ratio and angle of orientation of fiber in composites have almost the same impact on the Rockwell hardness number of the fabricated composites. Overall, to obtain the maximum hardness of the composite, it should be fabricated using 1:1 fiber ratio with 15° angle of orientation of the fiber in a single mat.

#### 3.4.2 Confirmation test for rockwell hardness

The highest Rockwell hardness is observed under A (1:1), B (15), and C (single mat) constraints. For the confirmation tests, three distinct samples were utilized, and the control parameters were adjusted to their ideal settings. The equation provided calculates the predicted maximum Rockwell hardness [38].

Rockwellhardness(Predicted)=A(1:1)+B(15)+C(Singlemat)–3Y

=57.56+56.67+64.89–3(51.14)

=25.7

At their optimal levels, Rockwell hardness number averages out to A, B, and C, with Y being the overall mean, for the maximum Rockwell hardness number.

Rockwell Hardness (Test) = 24.

Percentage of error (%) = 6.62 %

#### 3.4.3 Regression analysis for rockwell hardness

For the coir and PALF composites, the corresponding regression formula for Rockwell hardness is.

Rockwell hardness = 54.93–4.37 (A) - 0.26 (B)+ 9.96 (C)

**Fig. 22:**
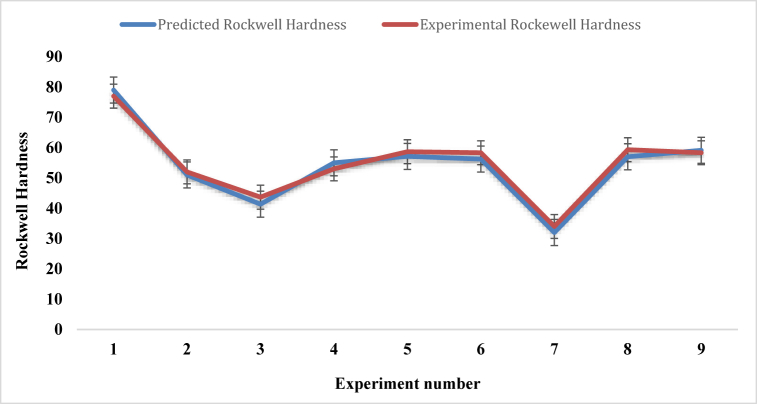
Rockwell hardness number comparison between experimental and predicted values.

Fig. 22 shows the graphical relationship between experimental Rockwell hardness number and predicted Rockwell hardness number. The two graphs in the figure have nearly identical natures. Every experimental Rockwell hardness number is almost assimilated with the predicted Rockwell hardness number except the experiment number of 3, 4, 6 and 8.

#### 3.4.4 ANOVA for rockwell hardness

Table-15 is the general linear model of ANOVA, in which the Rockwell hardness of the fabricated composites is primarily determined by the mat type with a maximum percentage contribution of 50.67 %. The F-distribution table yields the critical value F (critical) = 7.71 for numerator 1 and denominator 4. The highest contribution has a predicted F-value of 1.26, that is less than 7.71.0.442 or 44.2 % of P value is also higher than 5 % and suggests that the prediction is reasonable. On the other hand, the reinforced composite's angle of orientation displays the lowest level of contribution, with a value of 1.42 %. In this instance, the F value, 7.71, is not met by the predicted F-value of 0.04. Furthermore, the P-value is greater than 5 % at 0.996, or 99.6 %, suggesting a statistically significant way of accepting the prediction.

**Table-15:** ANOVA for 95 % confidence level of Rockwell hardness.

Source	DF	Adj SS	Adj MS	F-Value	P-Value	Contribution
Fiber ratio	2	87.21	43.606	0.19	0.838	7.78 %
Angle of orientation	2	15.89	7.943	0.04	0.966	1.42 %
Mat type	2	567.92	283.958	1.26	0.442	**50.67 %**
Error	2	449.70	224.850			40.13 %
Total	8	1120.71				100.00 %

### 3.5 Water absorption test

According to ASTM D5229M − 12, water absorption tests were conducted on coir and PALF composites by immersing them in stored rainwater at 25°, shown in Table-16. The sample composites were weighed on a regular basis of 24, 48, and 72 h consistently. The weight difference was used to calculate the water absorption rate.

**Table-16:** Average experimental water absorption rate under different control parameters.

Expt. No.	Fiber ratio	Angle of orientation	Mat-type	Water absorption rate (%)			
				Sample 1	Sample 2	Sample 3	Mean
1	1:1	30	Single mat	5.74	5.57	5.94	**5.75**
2	1:1	15	Combine Mat (cp-cp)	4.85	5.15	6.84	**5.61**
3	1:1	90	Combine Mat (cc-pp)	7.89	9.05	8.15	**8.36**
4	1:2	30	Combine Mat (cp-cp)	5.12	4.34	3.39	**4.28**
5	1:2	15	Combine Mat (cp-cp)	5.85	6.19	6.15	**6.06**
6	1:2	90	Single mat	8.47	9.71	10.5	**9.56**
7	2:1	30	Combine Mat (cc-pp)	2.29	2.62	5.96	**3.62**
8	2:1	15	Single mat	7.23	7.26	8.38	**7.62**
9	2:1	90	Combine Mat (cc-pp)	3.52	6.37	10.54	**6.81**

**Fig. 23:**
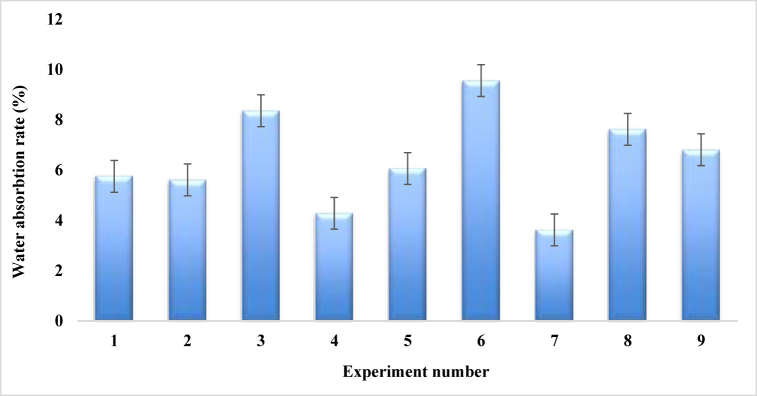
Comparison of water absorption rate for different experiment numbers.

Table-16 and Fig. 23 represent the percentage of water absorption obtained from the different experimental samples that were designed utilizing the Taguchi L9 method. The highest water absorption rate of 9.56 %, is found in experiment number 6, where the fiber ratio, angle of orientation, and mat type are expressed as 1:2, 90°, and single mat, respectively. The minimal percentage of water absorption value 3.62 %, is revealed by experiment number 7 which is better than the water absorption rate of Kevlar fiber reinforced composites [50], and bi-directional Typha angustifolia natural fiber reinforced composites [51], where the fiber ratio, angle of orientation, and mat type are respectively in 2:1, 30°, and combine mat (cc-pp). It is also noticed that, for the minimum water absorption by the composites, it should be fabricated with an angle of 30° orientation of the fibers that plays a major role in this case.

#### 3.5.1 Taguchi orthogonal array analysis for water absorption

Since the fundamental aim of this test is to minimize water absorption. Thus the “smaller is better” approach is applied in this method. Table-17 displays the mean water absorption rate at various control parameter levels.

**Table-17:** Response in mean water absorption rate at various levels of control factors.

Level	Fiber ratio	Angle of orientation	Mat type
1	6.57	6.43	7.64
2	6.63	**4.55**	**5.57**
3	**6.017**	8.24	6.01
Delta	0.617	3.69	2.07
Rank	3	1	2

**Fig. 24:**
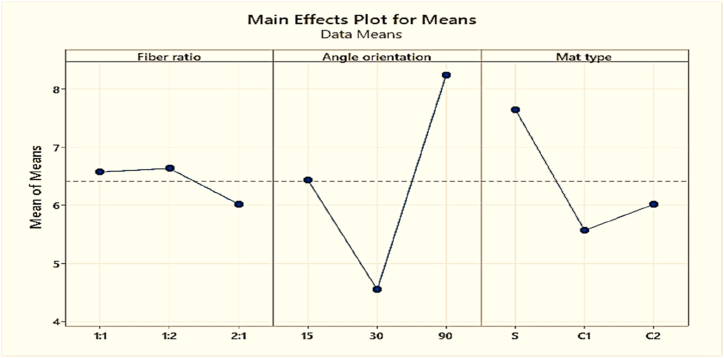
Composite's mean water absorption rate.

Fig. 24 demonstrates that maintaining the angle of orientation of the fibers to 90° is necessary to maintain a lower water absorption rate for the composite. Mat type is the second advantageous option for keeping the water absorption at a low level. For mat type S or single mat, the water absorption rate is low. The fiber ratio of coir fiber to pineapple leaf fiber is the third advantageous parameter for reducing water absorption. The composite's water absorption decreases as the weight percentage of pineapple fruit fiber increases.

#### 3.5.2 Confirmation test for water absorption

The optimal set of variables, according to the Taguchi design of experiments, is A (2:1), B (30), and C (combine mat). The approximate minimum water absorption can be calculated using the following formula [38]:

Waterabsorption(Predicted)rate=A(2:1)+B(30)+C(combinemat)–3Y

=(6.017+4.55+5.57)–(3x4.5)

=2.637%

At their optimal levels, water absorption averages out to A, B, and C, with Y being the overall mean, for the lowest proportion of water absorption.

Three new specimens were made because of the combination to support the predicted water absorption rate. The experimental water absorption rate was found to be 2.7 %. In this test, the percentage of error was 2.33 %, that is very much lower compared to 16.57 % [52].

#### 3.5.3 Regression analysis for water absorption

The regression equation for water absorption of the coir and PALF composites is:

WaterAbsorption=6.408+0.226(A)−1.858(B)+1.236(C)

**Fig. 25:**
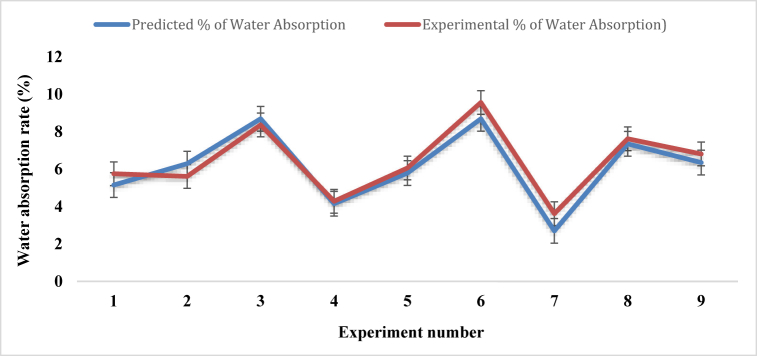
Water absorption rate comparison between experimental and predicted values.

Fig. 25 shows the graphical relationship between experimental and predicted water absorption rate. The two graphs in the figure have nearly identical natures. For experiment numbers 4, 5, and 8, the experimental percentage of water absorption values are almost assimilated with the predicted values. For experiment numbers 1, 2, 3, 6,7 and 9, the experimental values are very close to the predicted values.

#### 3.5.4 ANOVA for water absorption

Table-18 is the general linear model of ANOVA, in which the water absorption of the fabricated composites is primarily determined by the angle of orientation with a maximum percentage contribution of 70.89 %. The F-distribution table yields the F value of 7.71. The greatest percentage contribution has a projected F-value of 5.57, that is lower than the crucial F-value of 7.71. The significance level (P-value) is 0.326, or 32.6 %, that is more than 5 % and indicates that the prediction is plausible. Similarly, the reinforced composite's fiber ratio displays the lowest level of contribution, with a value of 2.40 %. In this instance, the F value of 7.71 doesn't match the predicted F-value of 1.28. Furthermore, the P-value is greater than 5 % at 0.839, or 83.9 %, suggesting a statistically significant likelihood of rejecting the assumptions. The combined impact of unaccounted-for control variables results in an error rate in this study of 1.88 %.

**Table-18:** ANOVA for 95 % confidence level of water absorption.

Source	DF	Adj SS	Adj MS	F-Value	P-Value	Contribution
Fiber Ratio	2	69.38	22.4	1.28	0.839	2.40 %
Angle of orientation	2	204.63	102.31	5.57	0.326	**70.89 %**
Mat type	2	134.90	35.8	3.22	0.570	24.83 %
Error	2	102.48	42.65			1.88 %
Total	8	600.49				100.00 %

## 4 Future recommendation

The recent work on PALF and coir fiber-based composites offers several avenues for further research into these materials. Here are a few proposals for further research.

i. This research can be further developed by incorporating various composite fabrication methods, including vacuum moulding or compression moulding.
ii. The humidity of natural fibre was not regulated in this study, as they were desiccated openly in sunlight following chemical treatment. The quality of the composite can be enhanced by a mechanical desiccator, which will regulate the humidity of the fibres. For this project, fibers were combined via epoxy and hardener employing a manual stirring mechanism. But using an electric mixing device can help to achieve a more uniform mixture.
iii. Other control variables may be considered, such as volume of fiber, degree of chemical treatment, mixture's temperature, manufacturing pressure, and the length of the curing period.
iv. The L25 (5^5^) orthogonal array is highly recommended for obtaining more accurate outcomes when optimizing the input parameters.

## 5 Conclusion

The following are the findings of - A Taguchi-Based Study on the Control Factors of Reinforced Composites with the Fiber of Coir and Pineapple Leaves.

• Based on the Taguchi orthogonal array analysis, it was observed that the mechanical characteristics were most significantly influenced by the type of mat and angle of orientation. The fibers in a single mat at a 15° angle of orientation exhibited exceptional impact and tensile strength, along with impressive Rockwell hardness. However, when the fibers were orientated at a 30° angle and combined into a mat, the flexural strength was maximized, and the water absorption rate was minimized. Although the impact of the wt.% of fiber ratio on the mechanical properties was not as significant, it remained an important factor in the production of the composites.
• A 1:2 fiber ratio showed the highest impact and flexural strength, while a 2:1 ratio was optimal for tensile strength and water absorption rate. A 1:1 ratio was found to be ideal for Rockwell hardness.
• After analyzing the experimental results obtained from the Taguchi L9 orthogonal method, it was discovered that there was margin of errors for various tests. The margin of errors was found to be 4.84 %, 2.59 %, 2.35 %, 6.62 %, and 2.334 % for impact, tensile, and flexural strength, Rockwell hardness, and water absorption rate respectively.
• The experimental and predicted results were compared using regression analysis. The results of the tests revealed that impact strength ranged between 2.93 and 0.4 J/cm^2^, tensile strength between 2.12 and 0.79 MPa, flexural strength between 3.54 and 0.33 MPa, the Rockwell hardness between 2.33 and 0.8 RHN, and percentage of water absorption test between 0.92 % and 0.13 %.
• The effects of several control parameters on impact, tensile and flexural strength, hardness, and water absorption test were evaluated using ANOVA analysis. The results indicated that in composites, the angle at which the fibers were orientated significantly affected the flexural strength and water absorption rate, accounting for approximately 72.30 % and 70.89 % respectively. However, it is worth noting that mat types had a significant impact on tensile strength and Rockwell hardness. Specifically, tensile strength was affected by approximately 46.47 %, while Rockwell hardness was affected by about 50.67 %. In addition, the impact strength was most significantly affected by the wt.% ratio of fibers, which was approximately 50.32 %.
• Due to biodegradability, recyclability, eco-friendly, and more economical materials, different types of mats, table linens, bags, and clothing items can be fabricated from these composite materials, which may remarkably take place the market of synthetic fiber based composite materials.

## CRediT authorship contribution statement

**Md Firoz Kabir:** Writing – review & editing, Writing – original draft, Supervision, Investigation, Conceptualization. **Md Alamgir Hossain:** Writing – review & editing, Writing – original draft, Validation, Formal analysis. **Md Nazmus Sakib:** Writing – original draft, Software, Methodology, Data curation, Writing – original draft, Software, Resources, Methodology, Data curation. **Md Waliullah Shadhin:** Writing – original draft, Software, Resources, Methodology, Data curation. **Md Ariful Alam:** Writing – original draft, Validation, Supervision, Formal analysis.

## Ethics declaration

Review and/or approval by an ethics committee as well as informed consent was not required for this study because this article did not involve any direct experimentation/studies on living beings.

## Data availability statement

The original contributions presented in the study are included in the article, further inquiries can be directed to the corresponding authors.

## Funding

This research received no external funding.

## Declaration of competing interest

The authors declare that they have no known competing financial interests or personal relationships that could have appeared to influence the work reported in this paper.
